# The Effect of S-Adenosylmethionine on Cognitive Performance in Mice: An Animal Model Meta-Analysis

**DOI:** 10.1371/journal.pone.0107756

**Published:** 2014-10-27

**Authors:** Sarah E. Montgomery, Amir A. Sepehry, John D. Wangsgaard, Jeremy E. Koenig

**Affiliations:** 1 Department of Applied Human Nutrition, Mount Saint Vincent University, Halifax, Nova Scotia, Canada; 2 College for Interdisciplinary Studies, Graduate program in Neuroscience, University of British Columbia (UBC), Vancouver, BC, Canada; 3 UBC Division of Neurology, Department of Medicine, University of British Columbia (UBC), Vancouver, BC, Canada; 4 Faculty of Computer Science, Dalhousie University, Halifax, NS, Canada; Van Andel Institute, United States of America

## Abstract

**Background:**

Alzheimer's disease (AD) is the most frequently diagnosed form of dementia resulting in cognitive impairment. Many AD mouse studies, using the methyl donor S-adenosylmethionine (SAM), report improved cognitive ability, but conflicting results between and within studies currently exist. To address this, we conducted a meta-analysis to evaluate the effect of SAM on cognitive ability as measured by Y maze performance. As supporting evidence, we include further discussion of improvements in cognitive ability, by SAM, as measured by the Morris water maze (MWM).

**Methods:**

We conducted a comprehensive literature review up to April 2014 based on searches querying MEDLINE, EMBASE, Web of Science, the Cochrane Library and Proquest Theses and Dissertation databases. We identified three studies containing a total of 12 experiments that met our inclusion criteria and one study for qualitative review. The data from these studies were used to evaluate the effect of SAM on cognitive performance according to two scenarios: 1. SAM supplemented folate deficient (SFD) diet compared to a folate deficient (FD) diet and 2. SFD diet compared to a nutrient complete (NC) diet. Hedge's g was used to calculate effect sizes and mixed effects model meta-regression was used to evaluate moderating factors.

**Results:**

Our findings showed that the SFD diet was associated with improvements in cognitive performance. SFD diet mice also had superior cognitive performance compared to mice on an NC diet. Further to this, meta-regression analyses indicated a significant positive effect of study quality score and treatment duration on the effect size estimate for both the FD vs SFD analysis and the SFD vs NC analysis.

**Conclusion:**

The findings of this meta-analysis demonstrate efficacy of SAM in acting as a cognitive performance-enhancing agent. As a corollary, SAM may be useful in improving spatial memory in patients suffering from many dementia forms including AD.

## Introduction

As of 2013, approximately 44.4 million people suffer from dementia [1]. Alzheimer's disease is the most widely recognized and diagnosed form of dementia [2]. It is a neurodegenerative disease characterized by severe cognitive impairment, with learning, memory, and visuospatial abilities being three of the most prominent behavioral processes to deteriorate [3].

S-adenosylmethionine (SAM), has gained considerable attention for its enhancing effect on cognitive performance in both human and animal model research [4]–[9]. SAM is a naturally occurring compound in the human body that carries out numerous metabolic reactions. SAM plays an integral role as methyl donor in the metabolism of Methionine (Met) to homocysteine (Hcy). In this conversion, SAM regulates epigenetic processes via DNA methylation [10] that have been implicated in its efficacy for the treatment of depression, osteoarthritis and liver support in humans [7], [11], [12].

There have been a few promising human studies evaluating the effects of SAM on improving cognitive performance in patients with AD [7], [8], [12]. However, there has been more extensive research on SAM using AD mouse models [5], [13]–[15]. To date, many of the animal studies exhibit differences in methodology protocols between studies as well as conflicting results. These differences have presented challenges in drawing definitive conclusions on the efficacy of SAM in improving cognitive performance.

Animal models commonly used in AD research, as it relates to metabolism, are used to evaluate the relationship between nutrient status and gene expression. Two mouse models used in the studies included in this meta-analysis are the apolipoprotein E4 (APOE4) model and the methylenetetrahydrofolate reductase deficiency (MTHFR −/−) model [13]. These two models have been associated with a greater risk for AD development, particularly in combination with a folate deficient diet [16]. It has previously been established that SAM confers a positive effect on cognitive performance in these two genotypes under folate deficient conditions [11]. Alternatively, Apolipoprotein E2 and E3 genotypes present protective and neutral effects on risk of developing AD, respectively [9]. All of these models, combined, provide a comprehensive illustration of SAM's effect on cognitive performance across varying genotypic backgrounds.

The outcome of this research is particularly important in the consideration of treatments for other conditions such as cardiovascular diseases and low bone mineral density since these diseases have frequently been associated with high levels of plasma Hcy and corresponding hypomethylation [17]–[19]. The widespread theory that elevated Hcy and corresponding hypomethylation are risk factors for multiple diseases and disorders suggests that many populations may benefit from these findings.

The purpose of this research is to evaluate the effect of SAM as a novel nutraceutical treatment for halting the progression of cognitive decline in both healthy and AD mouse models through a meta-analysis research design. We hope to further substantiate the evidence that SAM can exhibit a restorative effect on cognitive performance by evaluating the performance of various mouse strains in the Y maze and Morris Water Maze (MWM). Both mazes are well-established tools for measuring spatial learning and memory in mice and widely recognized and used in AD research [20]. Specifically, the studies in this meta-analysis evaluated the effect of a folate deficient (FD) diet and a SAM supplemented folate deficient (SFD) diet on percent spontaneous alternations in the Y maze and latency to swim, swimming speed and number of annulus crossings in the MWM using healthy and transgenic mice. Firstly, we tested the hypothesis that SAM supplementation improves cognitive performance in mice fed a folate deficient diet. Secondly, we tested the hypothesis that S-adenosylmethionine can raise cognitive performance to the level of mice fed a nutrient complete (NC) diet.

## Methods

### Search criteria

The Preferred Reporting Items for Systematic Reviews and Meta-Analyses (PRISMA, 2009) search strategy flow chart and checklist were used as guides in identifying and selecting relevant studies [21]. MEDLINE, EMBASE, Web of Science, the Cochrane Library and Proquest Theses and Dissertations database were used to search for articles containing a combination of the following words: s-adenosylmethionine, adomet, cognitive, cognition, dementia and Alzheimer's. Following these searches, MeSH terms were used to further extend the search. Study selection was restricted to the English language and all relevant papers published up until April 2014 were included in the analysis. Refer to Table S2 for an example of a more detailed search summary.

Reference lists of the selected articles from the original search were reviewed for additional relevant papers. The journals, of the articles that were selected from the primary search, were manually searched as well. A search of Proquest Dissertations and Theses was included to ensure that any unpublished theses were identified for relevant use. Correspondence authors were contacted for unpublished studies when possible; however, no unpublished works were available to us for two reasons: 1. There were no unpublished results that met the inclusion criteria or 2. The authors preferred not to disclose their work until it was submitted for publication.

### Inclusion and Exclusion Criteria

The following criteria were used in the selection of studies for our meta-analysis: 1. All data must be of mouse model origin, 2. Studies must have a cognitive performance outcome as measured by the Y maze or MWM, which are commonly used tools in evaluating spatial learning/memory test in both mice and rats [20], 3. Studies were required to have both a SFD diet group and one or two of the following additional groups: a FD diet and/or a NC diet. Both healthy strains and transgenic mouse models of AD were used in the analysis (Table 1). The measure of interest was cognitive performance in the Y maze as measured by % of spontaneous alternations and latency, swim speed and number of annulus crossing as measured by the MWM.

**Table 1:** Characteristics of mouse studies used in meta-analysis and qualitative analysis.

Study	Intervention	Sample size	Mouse model	Duration of treatment (months)	Age (months)	Favored Group:	Quality assessment score (%)^F^
						1. FD vs SFD	
						2. NC vs SFD	
**1. Chan et al 2008A** A	1. NC diet	1. n = 7^C^	Normal (C57B/6)	1	9–12	1. SFD	62
	2. FD diet	2. n = 7^C^				2. SFD	
	3. SFD diet (100 mg/kg)	3. n = 7^C^					
**2. Chan et al 2008B** A	1. NC diet	1. n = 7^C^	ApoE −/−	1	9–12	1. SFD	62
	2. FD diet	2. n = 7^C^				2. SFD	
	3. SFD diet (100 mg/kg)	3. n = 7^C^					
**3. Chan et al 2008C** A	1. NC diet	1. n = 7^C^	ApoE2	1	9–12	1. SFD	62
	2. FD diet	2. n = 7^C^				2. SFD	
	3. SFD diet (100 mg/kg)	3. n = 7^C^					
**4. Chan et al 2008D** A	1. NC diet	1. n = 7^C^	ApoE3	1	9–12	1. FD	62
	2. FD diet	2. n = 7^C^				2. NC	
	3. SFD diet (100 mg/kg)	3. n = 7^C^					
**5. Chan et al 2008E** A	1. NC diet	1. n = 7^C^	ApoE4	1	9–12	1. SFD	62
	2. FD diet	2. n = 7^C^				2. SFD	
	3. SFD diet (100 mg/kg)	3. n = 7^C^					
**6. Chan et al 2008F** A	1. NC diet	1. n = 7^C^	Healthy, Aged (C57B/6)	1	2–2.5 (years)	1. SFD	62
	2. FD diet	2. n = 7^C^				2. SFD	
	3. SFD diet (100 mg/kg)	3. n = 7^C^					
**7. Chan et al 2008G** A	1. NC diet	1. n = 7^C^	MTHFR +/+	1	9–12	1. SFD	62
	2. FD diet	2. n = 7^C^				2. NC	
	3. SFD diet (100 mg/kg)	3. n = 7^C^					
**8. Chan et al 2008H** A	1. NC diet	1. n = 7^C^	MTHFR +/−	1	9–12	1. SFD	62
	2. FD diet	2. n = 7^C^				2. SFD	
	3. SFD diet (100 mg/kg)	3. n = 7^C^					
**9. Shea et al 2007A** A	1. NC diet	1. n = 12	ApoE4	1 month SAM fortification for group 3 started 2 weeks after experiment started	9–12	1. SFD	66
	2. FD diet	2. n = 6				2. SFD	
	3. SFD diet (100 mg/kg)	3. n = 6					
**10. Shea et al 2007B** A	1. NC diet	1. n = 16	Normal (C57B/6)	1 month SAM fortification for group 3 started 2 weeks after experiment started	9–12	1. No comparison	66
	2. No group	2. N/A				2. SFD	
	3. SFD diet (100 mg/kg)	3. n = 8					
**11. Tchantchou et al 2004A** A	1. NC diet	1. n = 11^D^	ApoE−/−	1	9–12	1. FD	58
	2. FD diet	2. n = 11^D^				2. NC	
	3. SFD diet (80 mg/kg)	3. n = 11^D^					
**12. Fuso et al 2012A** B	1. NC diet	1. n = 10	TgCRND8	3	3+3 weeks	Not included for quantitative analysis^E^	100
	2. FD diet	2. n = 10					
	3. SFD diet (400 µg/mouse)	3. n = 9					
**13. Fuso et al 2012B** B	1. NC diet	1. n = 8	129SV	3	3+3 weeks	Not included for quantitative analysis^E^	100
	2. FD diet	2. n = 15					
	3. SFD diet (400 µg/mouse)	3. n = 12					

A Y maze used to measure spatial memory outcome.

B Morris Water Maze (MWM) used to measure spatial memory outcome.

C Sample size reported in original article as 3–4 mice in 2 independent experiments.

D Sample size reported as 3–4 mice/diet/experiment×2–4 experiments for a total of 6–16 mice per treatment group.

E Data not included in quantitative analysis due to differences in measures, included in qualitative analysis for discussion.

F Quality assessment conducted using the Gold Standard Publication Checklist by Hooijmans (2010); quality assessment scores re-scaled such that the study with the highest quality score was set at 100 (% scale).

### Data extraction and management

Two researchers independently reviewed and selected studies based on the inclusion criteria. Mouse strains, ages, diets/interventions, duration of intervention and cognitive outcomes were identified and extracted from the methods sections of each study (Table 1). Authors were contacted for original data when possible. Means and standard errors of Y maze percent alternation scores were extracted from bar graphs using GraphClick 3.0.2 if authors preferred not to share data. Standard errors were converted to standard deviations for calculation of effect sizes. MWM data was not subject to quantitative data extraction as this data was used as a qualitative contribution in comparing meta-analyses results.

### Study Quality Assessment

A modified version of the gold standard publication checklist (GSPC) developed by Hooijmans et al (2010) was used to assess the quality of the studies included in this analysis [22]. It should be noted that all studies, with the exception of Fuso et al 2012, were published prior to the publication of the GSPC; the GSPC was developed in response to insufficient detail provided in publications. The GSPC is a comprehensive and high quality tool for assessing quality of studies; however, its use in evaluating the quality of papers written prior to its publication may not be appropriate. Scores were re-scaled such that each study was ranked in comparison with the Fuso et al 2012 paper (the highest ranked paper in the overall analysis); Fuso et al 2012 was scaled to 100%.

### Statistical Analysis

Comprehensive Meta Analysis (Version 2.0) was used to analyze the data and generate forest plots and funnel plots [23]. Means and standard deviations were used to generate the effect size estimates (i.e., Hedge's g). The Hedges's g effect-size estimate was generated given that it adjusts for the variation in sample sizes [24]. A random effects model was used to summarize the data; random effects models account for methodological and in part statistical variability. Tests of heterogeneity including the Q-statistic and I^2^ statistic were calculated; I^2^ scores of 25%, 50%, and 75% were considered low, moderate, and high heterogeneity, respectively [25]. Heterogeneity was further investigated using categorical data variables including type of mouse model (healthy vs. transgenic) and specific genotypes (ApoE4). Mixed-effect model meta-regression was utilized to examine the effect of potential *a priori* selected moderating factors including age, duration of treatment, and study quality assessment, on the overall effect-size estimate [26]. Funnel plots were used to examine the overall heterogeneity via visual presentation.

## Results and Discussion

### Search Results

A total of 2563 articles were found across all searches combined (Figure S1). A total of 1037 unique titles remained, after duplicates were removed. After reviewing article titles and/or abstracts, 19 articles were selected for further review. After full text review, 14 articles were eliminated because there was no cognitive performance outcome included in the study design or because the study did not incorporate a SAM intervention. Five articles remained, three were used in the meta-analysis, one was included as a qualitative accompaniment in the discussion of the results (Fuso et al 2012) and one was eliminated on the basis of previously published results (Tchantchou et al 2006). The Fuso et al 2012 paper was excluded from the quantitative analysis because the learning/memory tests were different and had different scoring outcomes; these discrepancies would have led to increase methodological heterogeneity. The cognitive performance tasks in the Fuso study measured latency, swim speed and annulus crossings on the Morris water maze; neither of these three measures could be combined with the other studies in a quantitative analysis without disrupting the integrity of the results. Data from the Tchantchou et al 2006 study came from the same sample as data in the Tchantchou et al 2004 study. The Tchantchou et al 2006 publication was excluded because it did not contain Y maze data; therefore, the Tchantchou et al 2004 paper was used for its Y maze data.

A total of 3 studies including 12 experiments comprised of 12 complete diet groups, 11 deficient diet groups and 11 deficient+SAM groups were included in the meta-analysis (Table 1). The following sample sizes represent the summative sample size for each group: control (n = 100), FD (n = 84) and SFD (n = 81). Despite a low, but sufficient number of studies included in this meta-analysis, the number of mouse experiments contributed by these three studies made for sufficient power to conduct this meta-analysis. Finally, two studies including 4 subgroups comprised of 4 complete diet groups, 4 deficient diet groups and 2 deficient+SAM groups were included for qualitative review.

### Study Characteristics

The mice receiving a SAM supplemented diet were treated with 80–100 g/kg of SAM or a nutraceutical formula containing 80–100 g/kg of SAM (Table 1). Duration of treatment ranged from 2 weeks to 1 month and mice were between 9 and 12 months of age except for one group, which were 2–2.5 years of age [13]. All studies utilized a folate deficient framework to assess the effects of SAM on cognitive performance. The deficient diet in two studies also included a vitamin E deficiency and addition of iron as a pro-oxidant [13], [27]. Further to this, two of the studies used a nutraceutical formula comprised of a blend of several compounds including SAM, all of which have demonstrated to have neuroprotective properties [14], [15]. Table 1 summarizes the genotypes of the transgenic animals.

Chan et al (2008) and Tchantchou et al (2004) reported sample sizes as a range for each experiment (ie. 3–4 mice were used for each group in a single study). It was not possible to obtain individual sample sizes for each group from the authors and therefore, an average of the range was calculated for the sample size in order to obtain effect sizes. Publication bias was minimized by contacting authors for unpublished data; however, no further relevant data was identified following communication with authors.

### Effect of a SFD diet versus FD diet on cognitive performance

**Figure 1:**
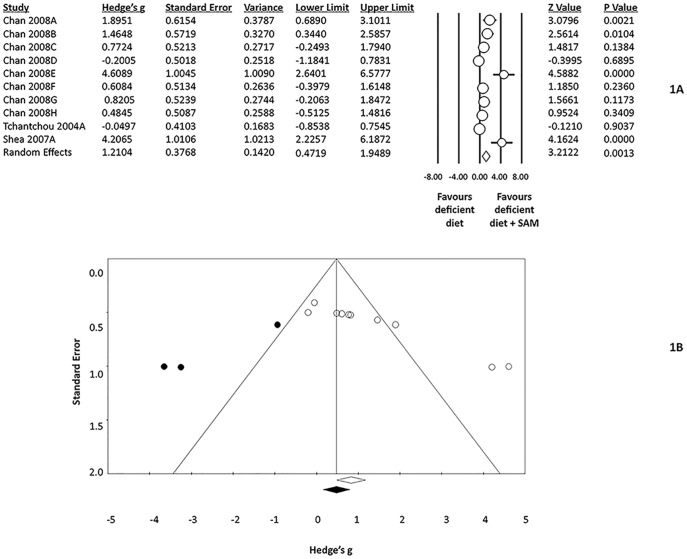
A) Effect size estimate: Comparison of a folate deficient diet and a folate deficient diet supplemented with S-adenosylmethionine (SAM) using Hedge's g as the effect size estimate statistic. B) Funnel Plot representation of potential publication bias in the comparison of a folate deficient diet supplemented with S-adenosylmethionine (SAM).

Three studies, including 10 subgroups, studied the effect of SAM supplementation on cognitive performance, within the context of a folate deficient diet. There was a significant effect of SAM supplementation on cognitive performance (Figure 1A, Table S3). Mice fed a SFD diet performed better than mice consuming a FD diet only (g = 1.2104[95%CI: 0.4719–1.9489], p = 0.0013). Heterogeneity was significant in the evaluation of the effects of a FD diet versus a SFD diet on cognitive performance (I^2^ = 76.9%, p<0.001). Healthy mice were analyzed separately from transgenic mice to further assess heterogeneity. In healthy mice, there was no difference between the groups (g = 0.9399[95%CI: −0.0184–1.8981], p = 0.0546). However, there was a significant effect of the SFD diet on cognitive performance in transgenic mice (g = 1.3272[95%CI: 0.3967–2.2577], p = 0.0052).

Heterogeneity was significant for the transgenic mouse analysis (I^2^ = 81.2%, p<0.001), but not for the healthy mouse analysis (I^2^ = 39.0%, p = 0.2003). The heterogeneity in the transgenic subgroup may be attributed to variations in performance response to FD diets across genotypes. There were three healthy mouse subgroups and seven transgenic mouse subgroups; therefore, the difference in sample size would no doubt contribute to differences in heterogeneity in the two analyses. There were not enough subgroups to apply genotype as a moderating variable to further evaluate heterogeneity; however, this would be a worthwhile analysis to consider in future meta-analyses when more data is available. Treatment effect size was associated with quality assessment (p<0.05) and treatment duration (p<0.05). Finally, a funnel plot of the 11 studies is presented in Figure 1B. The asymmetrical appearance of this plot suggests the potential presence of publication bias; however, it is difficult to assess this condition as none of these data come from unpublished work. With all studies coming from the same lab, there may or may not be an element of publication bias.

In comparison with results from the Fuso lab, there was a significant difference between the FD mice and the SFD mice in number of annulus crossings with the SFD group having a higher number of annulus crossings than the FD group. This result is in agreement with the Shea lab, in which, SAM supplementation improves cognitive performance in the Y maze in mice fed folate deficient diets. The increase in number of annulus crossings made by the TgCRND8 mice was not observed in wild type mice and, in fact, the opposite was observed, in which, mice consuming a SFD diet made fewer crossings than mice consuming a FD diet. The large differences between strains, across both labs, suggest substantial genotypic responses to folate deficiencies.

Quality assessment and diet duration both interacted with diet type (p<0.001 and p<0.001 respectively), accounting for a proportion of the variance in cognitive performance (Table S4). There was only one group of mice that differed in age from the other groups [13]; no association of age with diet type on cognitive performance was observed (p = 0.7533).

### SFD diet versus a NC diet and cognitive performance

**Figure 2:**
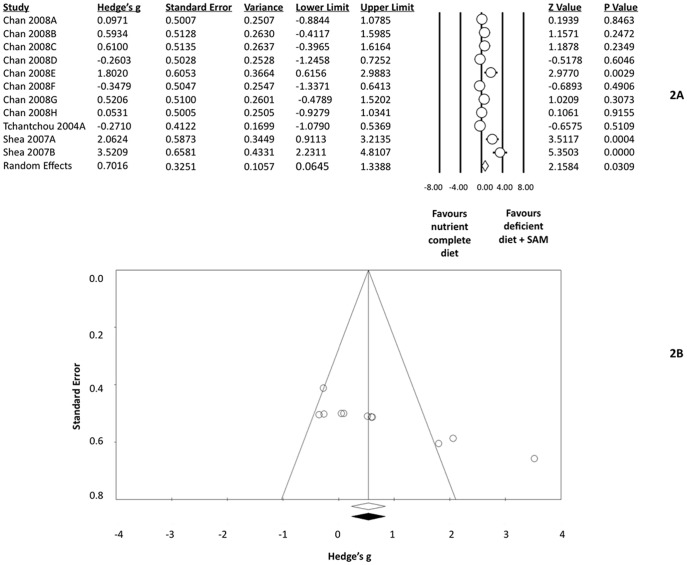
A) Effect size estimate: Comparison of a nutrient complete diet and folate deficient diet supplemented with S-adenosylmethionine (SAM) using Hedge's g as the effect size estimate statistic. B) Funnel Plot representation of potential publication bias in the comparison of a nutrient complete diet with a folate deficient diet supplemented with S-adenosylmethionine (SAM).

There was a significant difference between mice fed a SFD diet and mice consuming a NC diet (Figure 2A, Table S3). Mice fed a SFD diet performed better on the Y maze than mice fed a NC diet (g = 0.7016[95%CI: 0.0645–1.3388], p = 0.0309). Heterogeneity was significant (I^2^ = 76.7%, p<0.001) and an asymmetrical funnel plot suggested the possibility of publication bias (Figure 2B). None of the studies included in this analysis were unpublished; however, all efforts to contact authors were made. Therefore, we believe that publication bias is minimal.

Healthy animals and transgenic animals were analyzed separately to further investigate heterogeneity; despite separation of the groups, heterogeneity was still significant (Healthy: I^2^ = 89.4%, p<0.001; Transgenic: I^2^ = 65.9%, p = 0.0046). Healthy mice and transgenic mice, consuming the SFD diet, did not perform any better than mice consuming the NC diet for either group (Healthy: g = 1.3461[95%CI: −0.5743–3.2665], p = 0.1695; Transgenic: g = 0.4716[95%CI: −0.1357–1.0788], p = 0.1280). The heterogeneity in the healthy animal analysis may be explained, in part, by the substantial variability found among a small number of studies.

There was an association between diet type and quality assessment (p = 0.0116) and diet type and diet duration (p = 0.0164); however, there was no association between mouse age and diet type (p = 0.7533) on cognitive performance (Table S5). The impact of quality assessment and diet duration would inevitably contribute to the heterogeneity in this analysis. All of the mice from each subgroup were placed on their diets for 1 month except for the mice in the Shea (2007) study who were on diets for 2 weeks. Interestingly, the effect size for the Shea (2007) study was much greater than effect sizes for the other studies despite the 2-week diet duration; however, the sample size for the Shea 2007 study was much greater than the other studies and may partially explain this result (refer to table 1 for sample sizes).

In considering these findings, it is important to recognize that while all of the mice in the treatment group received SAM, one of the mouse groups received a nutraceutical formula containing SAM combined with other compounds; Shea (2007) included N-acetyl cysteine and acetyl-L-carnitine in their SAM-containing nutraceutical preparations. While SAM was the intervention of interest in this analysis, it is possible that other compounds in the formulation may have contributed to the positive result found in this analysis via an addition or synergistic mechanism. In addition, although all diets studied SAM supplementation within the context of a folate deficient diet, the Fuso lab deficient diet was also deficient in vitamins B6 and B12. The differences in study design of these experiments may contribute to variability between study results and this should be taken into consideration in the interpretation of these results. The small number of studies, containing other ingredients besides SAM, did not allow for further analysis of nutraceutical compounds as a moderating variable. However, evaluating clinical significance and efficacy of these neuroprotective compounds would be a worthwhile investigation in future reviews.

Additionally, it is important to note that while every effort was taken to increase accuracy of reported sample sizes, this error cannot be overlooked in the interpretation of these results. Underreporting methodological details in animal model studies is prevalent and more recently, publication reporting guidelines and checklists have been developed in response to these issues [22], [28]. It is imperative that future studies include these details in published work, either in the body of the paper or as supplementary material. In addition, publication bias has been demonstrated to be substantial in animal model literature. Registering animal model studies has been suggested as a means of reducing publication bias in meta-analyses; however, at this time there is no policy concerning this matter [29].

Although the literature search was extensive, all of the studies that were selected for this analysis and review came from only two labs; therefore, the diversity of this research, at this time, is limited. However, this meta-analysis allows for comparison of results within the Shea lab and between the Shea and Fuso labs and demonstrates that although there are some differences among findings within and between the labs, there appears to be evidence to support the use of SAM as a nutraceutical treatment for cognitive decline. Given the findings of this analysis, further research is warranted, particularly at the randomized controlled trial stage. Although it was not possible to include the Fuso et al (2012) study in the quantitative analysis, the trend of improved cognitive performance associated with SAM supplementation continues throughout the Fuso lab. The results of this meta-analysis substantiate the claim that SAM can improve cognitive performance in mice with compromise cognitive abilities. However, the number of studies, sample size accuracy and heterogeneity are substantial limitations and should be addressed in future studies.

The findings of this analysis suggest that there is evidence that SAM can improve spatial memory and learning in both healthy and transgenic mice fed a nutrient deficient diet. In combination, with human data from the Shea lab [12], [13], [30] and others [7], [8], [31], it is reasonable to expect that SAM could benefit those with reduced folate status or genotypes at higher risk for AD development.

### Implications for clinical practice and research

Further research concerning the roles of SAM and folate in human cognition should consider the relationship between folate fortification and SAM. Folate fortification, in North America, has resulted in a substantial improvement in folate status in North Americans; however, it appears that the effect of folate fortification on homocysteine concentrations is still unclear [32]. There is some evidence that an increase in folate status is not associated with a reduction in homocysteine; however, it is difficult to make this conclusion since B12 and B6 also play key roles in clearing plasma homocysteine [32]. This evidence, in conjunction with the theory that homocysteine may have no direct role in cognitive performance, suggests that the relationship between folate and SAM are still unclear. If dysfunction of the homocysteine-methionine cycle is not the direct cause of cognitive decline, then it is plausible that SAM exhibits an effect on cognitive performance separate from this cycle, which likely involves more complex epigenetic mechanisms.

Despite folic acid fortification, many people struggle to maintain sufficient folate status due to nutrient-drug/nutrient-gene interactions or poor intake of folate fortified foods [33]. Individuals taking “anti-folate drugs” such as oral contraceptives, anti-convulsants and anti-cancer drugs may be at an increased risk for poor folate status and subsequent risk for cognitive decline later in life [34], [35]. If there is a substantial impact of folate on SAM and vice versa, in relation to cognitive performance, then these populations stand to benefit from this supplement. SAM may also be an appropriate alternative for people who are unable to absorb and/or metabolize B vitamins from food or supplements. Of particular importance is decreased absorption of vitamin B12 in the elderly [36].

In addition to the effects of nutrient-drug interactions on folate metabolism, the differences in the ApoE4 mice, between the treatment and control groups, is noteworthy. It would be interesting to evaluate the effects of SAM supplementation on CpG methylation in these mice compared to ApoE2, ApoE3 and MTHFR −/− mice. If it is true that Apoe4 mice respond more strongly to SAM supplementation than other genotypes, then SAM may be more appropriate in the prevention of cognitive decline in the ApoE4 genotype than other genotypes. Further research using mouse and human models are needed to capture a more comprehensive analysis of the effects of this gene variant on SAM and cognitive performance.

Future research should consider the effect of SAM supplementation on healthy individuals consuming a healthy diet and individuals with gene variants who also consume a healthy diet. Investigation into the effects of SAM on disease risk in individuals taking anti-folate medications is also warranted. Finally, evaluation of the clinical significance and effectiveness of SAM as a supplement for cognitive performance is also important.

### Authors' conclusions

Supplementing a folate deficient diet with SAM appears to improve performance in spatial memory tasks on the Y maze. These results can be observed in transgenic mice, but not in healthy mice, when analyzed separately. More data with clear and consistent methodologies are required to substantiate these claims. Finally, genotypes with a higher risk for AD, such as the ApoE4 mouse, demonstrated greater responses to SAM supplementation than other genotypes, suggesting an epigenetic affinity by SAM for this animal model.

## Supporting Information

Figure S1 **PRISMA search strategy flow diagram.**
(TIF)

Table S1 **PRISMA checklist.**
(DOC)

Table S2 **Effect size estimates: 1. FD diet versus SFD diet, 2. NC diet versus SFD diet (n = 3).**
(DOCX)

Table S3 **Meta-regression analysis: FD diet versus SFD diet (N represents number of mouse studies).**
(DOCX)

Table S4 **Meta-regression analysis: NC diet versus SFD diet (N represents number of mouse studies).**
(DOCX)

Table S5 **Full search strategy for Cochrane Review search.**
(DOCX)

Table S6 **Raw data file.**
(XLSX)
