# Supplementary figures and images for: The Effect of S-Adenosylmethionine on Cognitive Performance in Mice: An Animal Model Meta-Analysis
Source: PLoS One. 2014 Oct 27;9(10):e107756. doi: 10.1371/journal.pone.0107756 (PMC4210123; doi:10.1371/journal.pone.0107756)

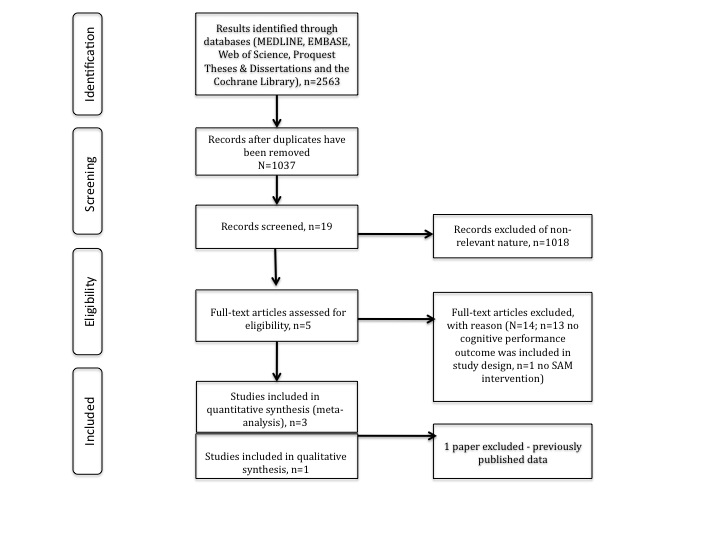

Supplement: Figure S1 — PRISMA search strategy flow diagram. (TIF) [file pone.0107756.s001.tif]
