# Supplementary material for: The Effect of S-Adenosylmethionine on Cognitive Performance in Mice: An Animal Model Meta-Analysis
Source: PLoS One. 2014 Oct 27;9(10):e107756. doi: 10.1371/journal.pone.0107756 (PMC4210123; doi:10.1371/journal.pone.0107756)
Supplement: Table S2 — Effect size estimates: 1. FD diet versus SFD diet, 2. NC diet versus SFD diet (n = 3). (DOCX) [file pone.0107756.s003.docx]

| Cochrane Review  (full search strategy with corresponding number of articles found in each search) | | |  |  |  |  |  |
| --- | --- | --- | --- | --- | --- | --- | --- |
|  |  |  |  |  |  |  |  |
| sam and cognition | sam and cognitive | sam and alzheimer's | sam and dementia | adomet and cognitive | adomet and cognition | adomet and dementia | adomet and alzheimer's |
|  |  |  |  |  |  |  |  |
| 5 | 9 | 2 | 3 | 0 | 0 | 0 | 0 |
