# Supplementary material for: The Effect of S-Adenosylmethionine on Cognitive Performance in Mice: An Animal Model Meta-Analysis
Source: PLoS One. 2014 Oct 27;9(10):e107756. doi: 10.1371/journal.pone.0107756 (PMC4210123; doi:10.1371/journal.pone.0107756)
Supplement: Table S3 — Meta-regression analysis: FD diet versus SFD diet (N represents number of mouse studies). (DOCX) [file pone.0107756.s004.docx]

| **Treatment Comparison** | | | **Treatment Effect** | | | | | **Heterogeneity** | | | |
| --- | --- | --- | --- | --- | --- | --- | --- | --- | --- | --- | --- |
| Folate deficient/Folate deficient + SAM | | |  |  |  |  |  |  |  |  |  |
| **Study** | **N (C/T)** | | **Hedge’s g** | **Lower limit** | **Upper limit** | **Z value** | **P value** | **Q** | **d.f.** | **p** | **I^2^** |
| Chan 2008 | 7/7 | | 1.8951 | 0.6890 | 3.1011 | 3.0796 | 0.0021 |  |  |  |  |
| Chan 2008 | 7/7 | | 1.4648 | 0.3440 | 2.5857 | 2.5614 | 0.0104 |  |  |  |  |
| Chan 2008 | 7/7 | | 0.7724 | -0.2493 | 1.7940 | 1.4817 | 0.1384 |  |  |  |  |
| Chan 2008 | 7/7 | | -0.2005 | -1.1841 | 0.7831 | -0.3995 | 0.6895 |  |  |  |  |
| Chan 2008 | 7/7 | | 4.6089 | 2.6401 | 6.5777 | 4.5882 | 0.0000 |  |  |  |  |
| Chan 2008 | 7/7 | | 0.6084 | -0.3979 | 1.6148 | 1.1850 | 0.2360 |  |  |  |  |
| Chan 2008 | 7/7 | | 0.8205 | -0.2063 | 1.8472 | 1.5661 | 0.1173 |  |  |  |  |
| Chan 2008 | 7/7 | | 0.4845 | -0.5125 | 1.4816 | 0.9524 | 0.3409 |  |  |  |  |
| Tchantchou 2004 | 11/11 | | -0.0497 | -0.8538 | 0.7545 | -0.1210 | 0.9037 |  |  |  |  |
| Shea 2007 | 6/6 | | 4.2065 | 2.2257 | 6.1872 | 4.1624 | 0.0000 |  |  |  |  |
| **Fixed** |  | | 0.8320 | 0.4885 | 1.1755 | 4.7476 | 0.0000 |  |  |  |  |
| **Random** |  | | 1.2104 | 0.4719 | 1.9489 | 3.2122 | 0.0013 | 39.0163 | 9.0000 | 0.0000 | 76.9327 |
| **Treatment Comparison** | | | **Treatment Effect** | | | | | **Heterogeneity** | | | |
| Complete/folate deficient + SAM | | |  |  |  |  |  |  |  |  |  |
| **Study** | | **N (C/T)** | **Hedge’s g** | **Lower limit** | **Upper limit** | **Z value** | **P value** | **Q** | **d.f.** | **p** | **I^2^** |
| Chan 2008A | | 7/7 | 0.0971 | -0.8844 | 1.0785 | 0.1939 | 0.8463 |  | | | |
| Chan 2008B | | 7/7 | 0.5934 | -0.4117 | 1.5985 | 1.1571 | 0.2472 |  |  |  |  |
| Chan 2008C | | 7/7 | 0.6100 | -0.3965 | 1.6164 | 1.1878 | 0.2349 |  |  |  |  |
| Chan 2008D | | 7/7 | -0.2603 | -1.2458 | 0.7252 | -0.5178 | 0.6046 |  |  |  |  |
| Chan 2008E | | 7/7 | 1.8020 | 0.6156 | 2.9883 | 2.9770 | 0.0029 |  |  |  |  |
| Chan 2008F | | 7/7 | -0.3479 | -1.3371 | 0.6413 | -0.6893 | 0.4906 |  |  |  |  |
| Chan 2008G | | 7/7 | 0.5206 | -0.4789 | 1.5202 | 1.0209 | 0.3073 |  |  |  |  |
| Chan 2008H | | 7/7 | 0.0531 | -0.9279 | 1.0341 | 0.1061 | 0.9155 |  |  |  |  |
| Tchantchou 2004 | | 11/11 | -0.2710 | -1.0790 | 0.5369 | -0.6575 | 0.5109 |  |  |  |  |
| Shea 2007A | | 12/6 | 2.0624 | 0.9113 | 3.2135 | 3.5117 | 0.0004 |  |  |  |  |
| Shea 2007B | | 16/8 | 3.5209 | 2.2311 | 4.8107 | 5.3503 | 0.0000 |  |  |  |  |
| **Fixed** | |  | 0.5400 | 0.2344 | 0.8457 | 3.4631 | 0.0005 |  |  |  |  |
| **Random** | |  | 0.7016 | 0.0645 | 1.3388 | 2.1584 | 0.0309 | 42.8440 | 10.0000 | 0.0000 | 76.6595 |
