# Supplementary material for: The Effect of S-Adenosylmethionine on Cognitive Performance in Mice: An Animal Model Meta-Analysis
Source: PLoS One. 2014 Oct 27;9(10):e107756. doi: 10.1371/journal.pone.0107756 (PMC4210123; doi:10.1371/journal.pone.0107756)
Supplement: Table S4 — Meta-regression analysis: NC diet versus SFD diet (N represents number of mouse studies). (DOCX) [file pone.0107756.s005.docx]

|  | N | Slope | SE | P-values |
| --- | --- | --- | --- | --- |
| Age | 10 | 0.2870 | 0.9131 | 0.7533 |
| QA/100 | 10 | 0.5775 | 0.2289 | 0.0116 |
| Duration of TX | 10 | -6.5106 | 2.7141 | 0.0164 |
