# Supplementary material for: The Effect of S-Adenosylmethionine on Cognitive Performance in Mice: An Animal Model Meta-Analysis
Source: PLoS One. 2014 Oct 27;9(10):e107756. doi: 10.1371/journal.pone.0107756 (PMC4210123; doi:10.1371/journal.pone.0107756)
Supplement: Table S5 — Full search strategy for Cochrane Review search. (DOCX) [file pone.0107756.s006.docx]

|  | N | Slope | SE | P-values |
| --- | --- | --- | --- | --- |
| Age | 11 | -0.0173 | 0.8478 | 0.9837 |
| QA/100 | 11 | 0.4676 | 0.1176 | 0.0001 |
| duration of tx | 11 | -6.1808 | 1.8356 | 0.0008 |
